# The Canadian Fire Spread Dataset

**DOI:** 10.1038/s41597-024-03436-4

**Published:** 2024-07-11

**Authors:** Quinn E. Barber, Piyush Jain, Ellen Whitman, Dan K. Thompson, Luc Guindon, Sean A. Parks, Xianli Wang, Matthew G. Hethcoat, Marc-André Parisien

**Affiliations:** 1 Northern Forestry Centre, Canadian Forest Service, Natural Resources Canada, 5320-122 Street NW, Edmonton, AB T6H 3S5 Canada; 2 Great Lakes Forestry Centre, Canadian Forest Service, Natural Resources Canada, 1219 Queen Street, Sault Ste. Marie, ON P6A 2E5 Canada; 3 Laurentian Forestry Centre, Canadian Forest Service, Natural Resources Canada, 1055 Rue du Peps, P.O. Box 10380, Station Sainte-Foy, Quebec City, QC G1V 4C7 Canada; 4 USDA Forest Service, Rocky Mountain Research Station, Aldo Leopold Wilderness Research Institute, 790 E. Beckwith Ave., Missoula, MT United States of America

**Keywords:** Natural hazards, Fire ecology

## Abstract

Satellite data are effective for mapping wildfires, particularly in remote locations where monitoring is rare. Geolocated fire detections can be used for enhanced fire management and fire modelling through daily fire progression mapping. Here we present the Canadian Fire Spread Dataset (CFSDS), encompassing interpolated progressions for fires >1,000 ha in Canada from 2002–2021, representing the day-of-burning and 50 environmental covariates for every pixel. Day-of-burning was calculated by ordinary kriging of active fire detections from the Moderate Resolution Imaging Spectroradiometer and the Visible Infrared Imaging Radiometer Suite, enabling a substantial improvement in coverage and resolution over existing datasets. Day of burning at each pixel was used to identify environmental conditions of burning such as daily weather, derived weather metrics, topography, and forest fuels characteristics. This dataset can be used in a broad range of research and management applications, such as retrospective analysis of fire spread, as a benchmark dataset for validating statistical or machine-learning models, and for forecasting the effects of climate change on fire activity.

## Background & Summary

Wildfire science is undergoing a data revolution enabled by improvements in the resolution and coverage of satellite-based observations of wildfire^1^. There has been a steady increase in the number and quality of active-fire monitoring sensors, first and foremost being the Moderate Resolution Imaging Spectroradiometer (MODIS), available 2001-present^2^, and the Visible Infrared Imaging Radiometer Suite (VIIRS), available 2012-present^3^. These low-earth orbit systems capture active wildfire data several times daily with no cost to the end user, allowing scientists and wildfire managers to track wildfire ignitions and daily spread^4–7^. Historically, data access challenges and processing requirements have limited efforts to consolidate and use these large active fire datasets, but with the launch of open data platforms such as Google Earth Engine^8^ (GEE) and the NASA Fire Information for Resource Management System^9^ (FIRMS), these barriers have largely been removed.

Researchers have developed several automated fire-event-delineation algorithms based on active-fire detections, thermal anomalies detected by satellite observation (hereafter “hotspots”). For example, FRY^10^ is a global database of fire patches built on the novel combination of MCD14A1 hotspots^11^ (hereafter “MODIS hotspots”) and change-detection maps, which were built on the integration of Collection 6 MCD64A1 burned area^12^ (hereafter “MODIS burned area”) and FIRECCI41^13^. The Global Fire Atlas^14^ and GlobFire^15^ go beyond fire delineation by using MODIS burned area^12^ to identify daily fire perimeters, providing information such as daily fire size and estimates of fire front location and direction of fire spread. The Fire Events Delineation^16^ (FIRED) algorithm provides further improvement through automated temporal clustering of fire events, producing a continuous estimate of fire spread rate and direction for the conterminous United States. However, all four algorithms (FRY^10^, Global Fire Atlas^14^, GlobFire^15^, and FIRED^16^) are reliant on the change-detection algorithm of MODIS burned area^12^ instead of fire hotspot interpolation, and thus they are subject to a multi-day temporal uncertainty and a 500-m or coarser resolution. For example, when MODIS burned area^12^ products were compared against MODIS hotspots, 32% of all MODIS burned area pixels reported fire arrival three or more days after the first hotspot detection^12^. Newer global burned area mapping algorithms such as FIRECCI50^17^ and FIRECCI51^18^ have reported finer spatial resolution and accuracy of day-of-burning estimates over MODIS burned area^12^, but still suffer from temporal uncertainty, which reduces their usefulness for identifying daily weather conditions during fire spread.

Interpolation of day-of-burning or fire arrival time (hereafter “progression mapping”) can be used to answer complex ecological and fire management questions. Hotspots and hand- or GPS-delineated fire progressions from aerial and ground observations have long been an essential part of wildfire operational management^19^. However, near-real-time fire progression mapping from satellite observations is gradually being practical^20^, given that continuous aerial observations by aircraft are no longer necessary. The Fire Event Data Suite^7^ (FEDS) algorithm uses spatiotemporal aggregation of VIIRS hotspots with an alpha hull to automate fire progression mapping at a 12-hr timescale. However, to better address missing hotspots, most automated fire progression mapping estimates fire arrival date using interpolation as opposed to spatiotemporal aggregation^5^. When assessed against high-quality infrared fire burned area observations, pooling VIIRS and MODIS and interpolating using kriging or natural neighbour interpolation leads to the best estimates of fire arrival time^21^. Although these methods are limited to approximately daily-scale accuracy by the limited satellite overpass frequency, methodological and sensor-technology advancements suggest that fire progression mapping on a subdaily scale is becoming possible. For example, coarse-resolution subdaily fire mapping using the Geostationary Operational Environmental Satellites (GOES) is possible for the Continental United States^22^; however, the satellites’ high view angles and the products’ coarse spatial resolution and high omission errors limit their usefulness at Canadian latitudes.

Improved fire progression mapping represents an opportunity to address a variety of wildfire research questions. For example, progression mapping provides an estimate of the date and thereby the weather conditions under which each pixel burned, necessary information for modelling fire spread under various fire weather conditions^6,23,24^. When coupled with maps of bottom-up factors such as land cover and topoedaphic characteristics, progression mapping can serve as a foundation for improving the knowledge base of large-scale wildfire spread phenomena, with applications involving wildfire in unconventional settings, such as novel disturbances or treed peatlands. Given that over twenty years of satellite observations of wildfire are available, even relatively rare circumstances or phenomena such as wildland-urban interface fires and high-latitude fires constitute hundreds or thousands of gridded data points for model building.

Here we present the Canadian Fire Spread Dataset^25^ (CFSDS), an event-based daily fire progression dataset at a resolution of 180 m, built on the combination of high-precision wildfire boundaries and active fire detections from multiple sensors. The CFSDS provides a research-oriented fire growth dataset that is useful for applications such as building statistical and machine-learning fire spread models. In this first release we provide a collection of interpolated wildfire progression maps and associated environmental covariates for all wildfires larger than 1,000 ha that occurred in the forested areas of Canada from 2002 to 2021. Hotspots^9^ were identified with co-located wildfire perimeters from the National Burned Area Composite^26^(NBAC). We combined VIIRS and MODIS hotspots^9^ to produce daily-scale fire progression maps for each fire with at least 6 observed hotspots^6^. We attributed each burned point (i.e. 180-m pixel) with 50 environmental covariates such as daily weather, derived weather metrics (e.g. drought metrics), topography, and forest attributes. The final product is a dataset of 3,269 fires comprising 70,895 days of burning, each with an estimate of the daily fire spread distance and area of growth.

## Methods

**Fig. 1:**
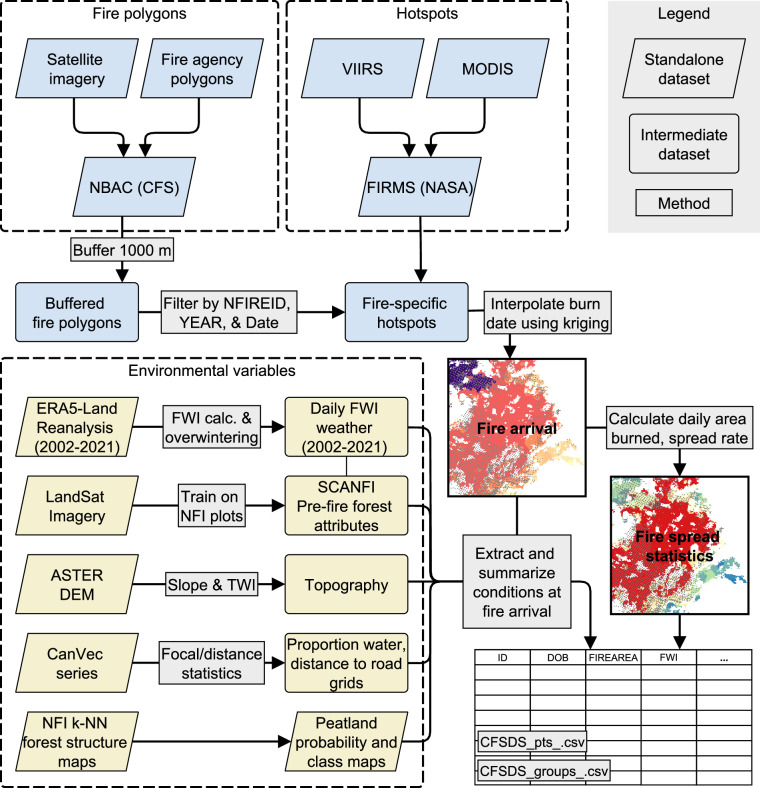
CFSDS production process. See Table 3 for covariate descriptions.

We constructed the CFSDS^25^ from three complementary groups of spatial and temporal data: final fire perimeter polygons, hotspots, and associated environmental covariates. In brief, hotspots were first filtered to predetermined fire perimeters. The detection times of these hotspots were then interpolated using ordinary kriging to provide an estimate of the date at which fire reached any given pixel within the fire perimeter. This raster of fire arrival times (i.e. “day of burning”) was then used to identify environmental covariates associated with the fire (Fig. 1).

### Fire event delineation and burned area polygons

**Fig. 2:**
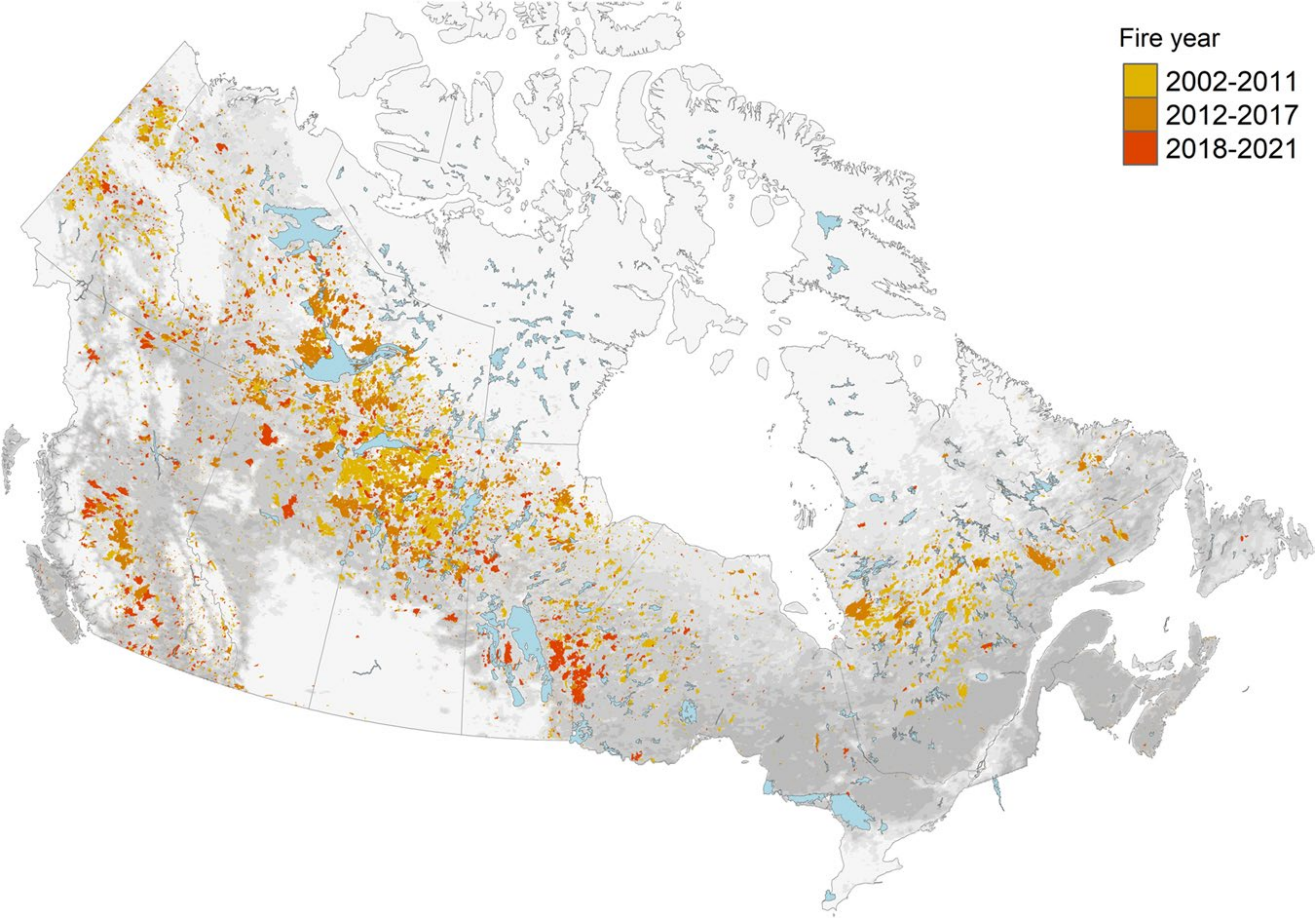
CFSDS fire polygons from NBAC. Fire year indicates the era of hotspot collection: 2002–2011 includes MODIS only, 2012–2016 includes MODIS and Suomi-NPP (VIIRS), 2017–2021 includes MODIS, Suomi-NPP, and NOAA-20 (VIIRS). Grey indicates mean canopy cover^48^.

We obtained wildfire perimeters from NBAC^26^, an annual, national fire dataset mapping individual wildfire events in Canada (Fig. 1). NBAC^26^ integrates three Canadian burned-area products, listed in order of decreasing precedence: pre- and post-fire Landsat imagery processed into normalized burn ratio using the Multi-Acquisition Fire Mapping System^27^ (MAFiMS); the Canadian National Fire Database^28^ (CNFDB), created from provincial/territorial wildfire agency polygon data; and the Hotspot and Normalised Difference Vegetation Index Differencing Synergy (HANDS) algorithm^29^. NBAC^26^ also includes non-spatial attributes for each fire, such as fire start date and fire end date, used here for subsetting hotspots. From the full NBAC^26^ record, we selected all fires that: (i) occurred from 2002–2021, and (ii) attained a final area of at least 1,000 ha (Fig. 2).

### Hotspots

We downloaded all MODIS and VIIRS hotspots within Canada from 2002 to 2021 from the NASA FIRMS^9^ (Fig. 1). The Collection 6 MODIS hotspots^2,30^ (MCD14ML, V006/V0061) includes all Terra (MOD14) and Aqua (MYD14) satellite detections at a spatial resolution of 1 km and four daily overpasses. VIIRS hotspots include both the Suomi-NPP^31^ (VNP14IMGT) and NOAA-20^32^ (VJ114IMGTDL_NRT) satellite detections at a spatial resolution of 375 m and four daily overpasses^3^.

In total, this consisted of 3,731,070 hotspots, including all pooled MODIS and VIIRS hotspots^9^. The temporal resolution of the data depends on the year it was collected, due to satellite launches: four times daily from 2002 to 2011, six times daily from 2012 to 2017, and eight times daily from 2018 to 2021. We filtered hotspots to individual fire events using NBAC^26^ polygon date and spatial location (Fig. 1). To account for hotspot precision limitations, all hotspots within 1,000 m of a fire polygon were considered for interpolation. Any hotspots occurring more than 30 days before the recorded fire start date or more than 30 days after the recorded fire end date were removed, to eliminate spatially coincident burning (from agricultural or silvicultural debris burning, for example) while still allowing for possible errors in fire start and end date attribution. We also applied a filter based on hotspot “confidence”, as reported by FIRMS^9^; MODIS hotspots with “confidence” less than or equal to 10 (range 0–100) and VIIRS hotspots with “confidence” of “LOW” (range “LOW” to “HIGH”) were removed, given that these hotspots are commonly associated with sun glint^9^. Hotspots were also adjusted to represent local standard time based on geographic location.

### Fire arrival time interpolation and processing

For each individual fire, fire arrival times were interpolated using kriging on a 180-m resolution base grid (Fig. 1). This resolution was selected to produce outputs that were a practical size for storage and upload, while still being a multiple of our covariate datasets’ resolution. Using all hotspots corresponding to an individual fire, we fit a variogram on hotspot detection times, as represented by the decimal numerical day of year (i.e. Feb. 1, 1200 hr as 32.5). We fit the experimental variogram using an exponential, spherical, and Matern model, and subsequently selected the best-fitting model for each fire. We used the default nugget, sill, and range values as determined by the *gstat* R package^33^. Using ordinary kriging, we interpolated the fire arrival time based on the fire-specific variogram fit. The resultant fire arrival time raster was then masked to burned areas as identified in NBAC^26^. The fire arrival date was rounded to the closest (temporally) date that was observed in the 6 nearest hotspots; this ensured that the predicted burning date occurred during detected fire growth and was not an artefact of averaging^6^.

The phenomenon of “peak burning” describes the tendency for wildfires in Canada to burn most intensely in the late afternoon or early evening^34^, a time of day during which the MODIS and VIIRS satellites are not present overhead. This artificially delays wildfire detection; for example, the VIIRS satellite overpasses occur at approximately 0100–0230 hr and 1300–1430 hr for the Canadian boreal zone. Therefore, the uncorrected day-of-burning rasters are biased towards late, nighttime detections, which may not always represent true fire arrival time. To limit the impact of these missing observations, we assigned any hotspot observed between 1900–0800 hr to the previous day. We assigned an observed time of 1900 hr to these hotspots, immediately after diurnal “peak burning” as determined by peak Fire Radiative Power (FRP) using GOES-16 and GOES-17^35^. In this case, the temporal rounding of hotspots (i.e. kriging inputs) is not to be confused with temporal rounding of the day-of-burning grid (i.e. kriging outputs).

#### Zero-growth days

Days with no observable fire growth were included in the dataset as zero-growth days, defined as any day with less than three pixels (9.72 ha) of fire growth. Environmental covariates were defined using the spatial location of pixels from the next day of non-zero fire growth (a “fire growth day”). The subsequent fire growth day was taken as the next sequential day in which the fire grew more than three pixels. False zero-growth days are possible, but less likely due to the high detection efficiency of nighttime active fire detections^30–32^.

### Environmental covariates

Daily fire arrival time interpolations were intersected with spatial datasets of 50 environmental covariates (Table 1) (Fig. 1), providing a record of the conditions under which each pixel burned. Environmental covariates were resampled to the same 180-m resolution grid used for interpolation of fire progression, where needed. We resampled using a mean aggregate function for continuous variables and a mode aggregate function for categorical variables.

#### Fire weather

We obtained weather data from the ERA5-Land dataset^36^, produced globally, hourly at 0.1 deg resolution. Specifically, 2-m air temperature, dewpoint temperature, total precipitation, 10-m u and v components of wind and snow cover variables were downloaded over Canada for the temporal period of the study (2002–2021). The data were further processed to provide the necessary inputs to the Canadian Forest Fire Weather Index (FWI) System^37^: the noon LST values of temperature, relative humidity, wind speed and the 24-hr accumulated precipitation ending at noon LST each day. The FWI System produces six indices: Fine Fuel Moisture Code (“FFMC”), Duff Moisture Code (“DMC”), Drought Code (“DC”), Initial Spread Index (“ISI”), and Buildup Index (“BUI”), and Fire Weather Index (“FWI”). The FWI System variables were calculated only for the snow-free period corresponding to the main fire season each year; season start was determined by the first day following three consecutive days of less than 50% snow cover. The DC is adjusted at startup by applying an overwintering correction based on the amount of overwinter precipitation^38^.

In addition to the FWI System outputs, the following daily weather variables were also included in the dataset: the maximum daily temperature (“Tmax”; °C), precipitation summed over 24 hr, from noon the prior day (“Prec”; mm), noon wind speed at 10-m elevation (“WS”; km/h), noon relative humidity (“RH”; %), and maximum vapour pressure deficit over a 24 hr period (“VPD”; hPa). Fire weather data is provided for all fires at the CFSDS data repository^25^.

#### Fuels

We represented modeled pre-fire fuels with the Spatialized Canadian National Forest Inventory data product^39^ (SCANFI). In brief, Landsat multispectral imagery was trained on national forest inventory plots and used to map pre-fire fuels at a 30-m resolution, including percentage deciduous component (“prcD”), percentage coniferous component (“prcC”), biomass in tonnes/ha (“Biomass”), and crown closure percentage (“Closure”).

#### Topography

All topographic variables were derived from the NASA ASTER Global Digital Elevation Model V003^40^ 30-m digital elevation model (“DEM”). This DEM was subsequently used to calculate elevation (“elev”), slope (“slope”), aspect (“aspect”), and topographic wetness index (“TWI”).

#### Peatlands and surface water

Peatland presence (“peatland”) and peatland class (“peatCl”) are the modeled probability that any pixel is a peatland, and the modeled peatland type from 9 possible classes (e.g., bog, fen, etc.) (Table 1). These estimates were based on the combination of MODIS reflectance data trained on National Forest Inventory plots to estimate the distribution of treed and open peatlands at a resolution of 250 m, supplemented with Landsat-based land-cover datasets^41^.

**Table 1:** Peatland classes^41^ used in “peatCl” covariate.

Peatland class	peatCl code
No peatland	0
Open bog	1
Open poor fen	2
Open rich fen	3
Treed bog	4
Treed poor fen	5
Treed rich fen	6
Forested bog	7
Forested poor fen	8
Forested rich fen	9

Proportion of surface water (“hydrodens”) within a 2 km, 5 km, 10 km, and 25 km of each pixel was calculated using the 50 K CanVec Series Hydrographic Features dataset^42^.

#### Anthropogenic

We represented road densities (“roaddens”) and distance to road (“roaddist”) using the 50 K CanVec Transport Features dataset^43^. Mean road density within 2 km, 5 km, 10 km, and 25 km of each pixel was calculated as the total length of roads per km^2^. Distance to road is based on linear distance to major roads or rail lines, not including temporary roads such as logging or access roads.

#### Fire characteristics

We calculated fire daily growth characteristics from the day-of-burn raster (*NFIREID_YEAR_krig.tif*). This includes daily area burned in hectares (“firearea”), cumulative area burned (“cumuarea”), and percent growth over the prior day’s total area (“pctgrowth”). The day since the start of fire (“fireday”) was calculated until extinguishment, including zero-growth days. Fire spread (metres per day) was estimated from the cumulative area burned relative to the cumulative area burned for the previous day. This assumes circular growth in a constant direction, according to the Eq. 1:

1 $$sprdistm=2(\sqrt{cumuarea/\pi }-\sqrt{(cumuarea-firearea)/\pi })$$

using area in m^2^. This calculation is an approximation of frontal fire spread, and not true linear spread^44^. This is an approximation of spread distance that has lower precision than the underlying hotspot data, and this value is not rounded based on the underlying resolution of hotspot data.

#### Ecozones

Ecozones, which are large geographic areas sharing biophysical characteristics, were identified from the National Ecological Framework for Canada^45^ (Table 2). The Boreal Shield and Taiga Plains ecozones have been split into “East” and “West” subzones^46^.

**Table 2:** Ecozone names and associated “ecozone” code^45^.

Zone name	“ecozone” code
Arctic Cordillera	1
Northern Arctic	2
Southern Arctic	3
Taiga Plains	4
Taiga Shield West	5
Boreal Shield West	6
Atlantic Maritime	7
Mixedwood Plains	8
Boreal Plains	9
Prairies	10
Taiga Cordillera	11
Boreal Cordillera	12
Pacific Maritime	13
Montane Cordillera	14
Hudson Plains	15
Taiga Shield East	25
Boreal Shield East	26

## Data Records

The complete CFSDS^25^ for 2002–2021 is provided open access at the Centre for Open Science OSF data repository (10.17605/OSF.IO/F48RY). This includes two datasets for quantitative analysis and one collection of fire arrival time interpolations:

*Firegrowth_pts_v1_YEAR.csv –* a data table where each row indicates one pixel (180 m) that burned on a specific day. Each row representing a pixel is attributed with the 50 covariates.

*Firegrowth_groups_v1.csv –* a summarised data table where each row indicates a single fire growth day. Environmental covariates are summarised over each specific day of a fire by taking the mean value of continuous covariates and the mode of categorical covariates. Latitude and longitude values represent the centroid of fire spread on each specific day.

*YEAR_NFIREID_krig.tif* – raster dataset depicting fire arrival time for a single fire, with values indicating the day of year at which the fire arrived in any given 180-m pixel. For example, a pixel burned on February 10 would have an integer value of 41. NFIREID and YEAR are replaced with attributes as identified in NBAC^26^. Rasters are grouped by year for download.

In the *Firegrowth_pts* and *Firegrowth_groups* datasets, data columns indicate the covariates under which the fire burned, by pixel and fire growth day respectively (Fig. 1). These variables are listed in Table 3. These datasets are intended for use in model building and statistical analysis.

**Table 3:** Environmental covariates and other associated fields.

attribute	description	mean ± sd	source
ID	Fire ID	NA	NBAC^26^
DOB	Day of year burning	203 ± 28	CFSDS^25^
year	Year	2012 ± 5.7	NBAC^26^
fireday	Day of fire (ignition day = 1)	21.9 ± 21.1	CFSDS^25^
firearea	Fire growth this day (ha)	571 ± 2383	CFSDS^25^
ecozone	Ecozone (see Table 4)	NA	ESWG (1996)^45^
fwi	Fire weather index	11.1 ± 9.7	CFSDS^25^, ERA5-Land^36^
isi	Initial spread index	4.1 ± 3.4	CFSDS^25^, ERA5-Land^36^
ffmc	Fine fuel moisture code	79.0 ± 15.8	CFSDS^25^, ERA5-Land^36^
dmc	Duff moisture code	36.7 ± 26.5	CFSDS^25^, ERA5-Land^36^
dc	Drought code	285 ± 127	CFSDS^25^, ERA5-Land^36^
bui	Buildup index	51.6 ± 31.4	CFSDS^25^, ERA5-Land^36^
prec	24-hr precipitation (mm)	1.5 ± 3.2	ERA5-Land^36^
vpd	Daily max vapour pressure deficit (hPa)	15.8 ± 6.4	ERA5-Land^36^
tmax	Maximum daily temperature (°C)	20.9 ± 4.9	ERA5-Land^36^
ws	Noon windspeed at 10 m elevation (km/h)	9.4 ± 4.7	ERA5-Land^36^
rh	Noon relative humidity (%)	45.8 ± 11.9	ERA5-Land^36^
fwi_prev1	Fire weather index from previous day	11.0 ± 9.6	CFSDS^25^, ERA5-Land^36^
fwi_prev2	Fire weather index from 2 days prior	10.9 ± 9.6	CFSDS^25^, ERA5-Land^36^
rh_prev1	Noon relative humidity from previous day (%)	47.3 ± 12.6	ERA5-Land^36^
rh_prev2	Noon relative humidity from 2 days prior (%)	48.4 ± 13.3	ERA5-Land^36^
d_fwi	Fire weather index anomaly	5.1 ± 8.8	CFSDS^25^, ERA5-Land^36^
d_isi	Initial spread index anomaly	1.4 ± 3.2	CFSDS^25^, ERA5-Land^36^
d_ffmc	Fine fuel moisture code anomaly	7.8 ± 15.7	CFSDS^25^, ERA5-Land^36^
d_dmc	Duff moisture code anomaly	16.4 ± 22.3	CFSDS^25^, ERA5-Land^36^
d_dc	Drought code anomaly	83.7 ± 70.5	CFSDS^25^, ERA5-Land^36^
d_bui	Buildup index anomaly	21.6 ± 25.5	CFSDS^25^, ERA5-Land^36^
d_prec	Precipitation anomaly (mm)	−1.0 ± 3.2	ERA5-Land^36^
d_vpd	Daily max vapour pressure deficit anomaly (Pa)	6.5 ± 4.7	ERA5-Land^36^
d_tmax	Maximum daily temperature anomaly (°C)	6.0 ± 4.5	ERA5-Land^36^
d_ws	Noon windspeed anomaly (km/h)	2.2 ± 4.2	ERA5-Land^36^
d_rh	Noon relative humidity anomaly (%)	6.5 ± 21.8	ERA5-Land^36^
Biomass	Total live aboveground biomass (t/ha)	53 ± 46	SCANFI^39^
Closure	Crown closure (%)	41 ± 16	SCANFI^39^
prcD	Deciduous component (%)	11 ± 13	SCANFI^39^
prcC	Conifer component (%)	89 ± 13	SCANFI^39^
peatCl	Peatland class	NA	Thompson *et al*.^41^
peatland	Modeled probability of peatland presence (0–100)	29 ± 19	Thompson *et al*.^41^
hydrodens2k	Proportion surface water within 2 km window	0.09 ± 0.12	CanVec Hydro^40^
hydrodens5k	Proportion surface water within 5 km window	0.11 ± 0.12	CanVec Hydro^40^
hydrodens10k	Proportion surface water within 10 km window	0.12 ± 0.12	CanVec Hydro^40^
hydrodens25k	Proportion surface water within 25 km window	0.13 ± 0.12	CanVec Hydro^40^
roaddens2k	Road density within 2 km (km/km^2^)	0.03 ± 0.12	CanVec Transport^43^
roaddens5k	Road density within 5 km (km/km^2^)	0.03 ± 0.10	CanVec Transport^43^
roaddens10k	Road density within 10 km (km/km^2^)	0.03 ± 0.10	CanVec Transport^43^
roaddens25k	Road density within 25 km (km/km^2^)	0.03 ± 0.09	CanVec Transport^43^
roaddist	Distance to major road or railway line (m)	39000 ± 39180	CanVec Transport^43^
dem	Elevation (m)	557 ± 384	ASTER Global Digital Elevation^40^
slope	Slope (degrees)	5.0 ± 6.4	ASTER Global Digital Elevation^40^
twi	Topographic wetness index	5.6 ± 0.5	ASTER Global Digital Elevation^40^
aspect	Aspect (degrees, 0–359)	181 ± 103	ASTER Global Digital Elevation^40^
cumuarea	Cumulative fire area (ha, NBAC^26^)	14450 ± 39510	CFSDS^25^
pctgrowth	Percent growth this day (%) (NBAC^26^)	113 ± 1950	CFSDS^25^
prevgrow	Fire growth on prior fireday (ha)	617 ± 2463	CFSDS^25^
sprdistm	Daily fire spread (m)	453 ± 1063	CFSDS^25^
lat	Latitude (m) (EPSG 4269)	−112 ± 16	CFSDS^25^
lon	Longitude (m) (EPSG 4269)	58 ± 5	CFSDS^25^

## Technical Validation

**Fig. 3:**
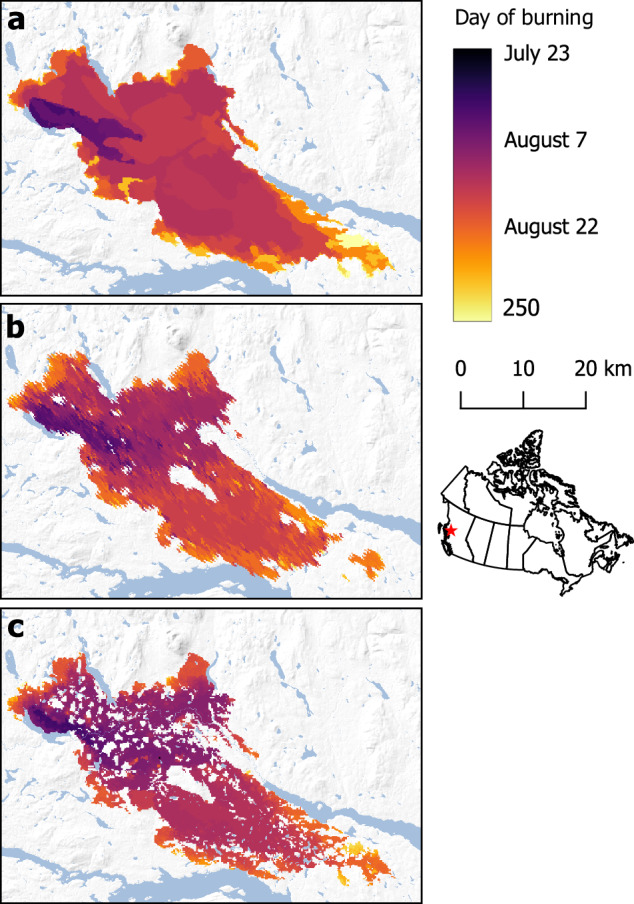
Estimated fire arrival time for the 2018 Nadina Lake Fire in British Columbia. Fire arrival estimated from the agency polygons (**a**), FIRED (**b**), and CFSDS (**c**).

Validating day-of-burning rasters is difficult because scant “ground truth” validation data is available, making it hard to assess with confidence the accuracy of our day-of-burn rasters. The best data available are fire progression perimeters from wildfire agencies (“agency perimeters”); however, the regular production of agency polygons is limited by resource availability and they are known to slightly underpredict fire arrival time^6^. Therefore, instead of quantifying absolute error, we validated CFSDS^25^ perimeters by assessing the degree to which they were an improvement over daily FIRED^16^ progressions for all of Canada, when compared against agency perimeters (Fig. 3). FIRED^16^ is the most recent and precise automated fire arrival time dataset with global coverage, compared against FRY^10^, Global Fire Atlas^14^ or GlobFire^15^. Where all three datasets (FIRED, CFSDS^25^, and agency perimeters^16^) were available we assessed CFSDS and FIRED against the agency perimeters using two methods: daily perimeter similarity using Sørensen-Dice index and daily area-burned correlation. Wildfire agency perimeters were provided by the provinces of British Columbia (84 fires, n = 497), Alberta (18 fires, n = 96), Saskatchewan (13 fires, n = 31), Manitoba (17 fires, n = 59), and Quebec (12 fires, n = 58).

### Preprocessing

The three datasets were substantially different and it was therefore necessary to subset them for a fair comparison. First and most importantly, the agency perimeters vary substantially in quality, including high-quality sources such as infrared aerial platforms and medium- or low-quality sources such as aerial GPS observations of fire perimeters. We only used agency perimeters with higher spatial and temporal precision as a validation dataset, defined as perimeters derived from: aerial photography; GPS aerial observations; processed infrared aerial imagery; Sentinel-2 satellite observations; and Landsat satellite observations. Both CFSDS^25^ and FIRED^16^ were subset to include only perimeters from dates which had representative observations in the agency polygons.

Second, CFSDS^25^ is unfairly advantaged against FIRED^16^ because CFSDS is constrained with an *a priori* fire perimeter, whereas FIRED is not. CFSDS^25^ often matches the final agency perimeter very closely due to similar data sources being used for fire delineation in NBAC^26^; therefore, we excluded the final perimeter as a data point. Instead all validation stats were calculated on fire progression polygons from the period while the fire was still expanding and we compared area burned as a percentage of final perimeter size instead of absolute area burned. Using percentage area burned prevents FIRED^16^ from being penalised by a high false-negative rate due to the lack of an *a priori* perimeter. Finally, to further reconcile the three datasets, we introduced unburned islands to FIRED^16^ and the agency perimeters by masking them with the fire NBAC^26^ polygon, as was done for CFSDS^25^.

### Perimeter similarity using Sørensen–Dice index

The Sørensen–Dice index is broadly used in image segmentation and more broadly to examine classification problems. It is equal to twice the area of overlap between perimeters divided by the total number of pixels in both perimeters (Eq. 2).

2 $${\rm{DSC}}=\frac{2TP}{2TP+FP+FN}$$

Where TP is true positive, FP is false positive, and FN is false negative. The index varies from 0 to 1, with 0 indicating no spatial alignment and 1 indicating perfect spatial alignment. We assessed perimeter similarity by calculating the Sørensen–Dice index between CFSDS-Agency and FIRED-Agency pairs for the same date. We used this index as an indicator of mean absolute improvement of CFSDS^25^ over FIRED^16^.

**Fig. 4:**
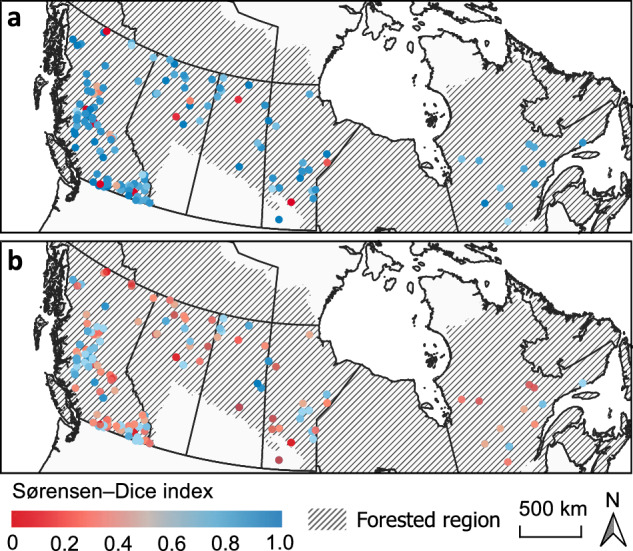
Sørensen–Dice index of agency polygons compared against CFSDS (**a**) and FIRED (**b**) polygons. Each point represents the mean index of a single fire, averaged across all days of burning.

Validation by Sørensen–Dice index indicates that CFSDS^25^ agrees more closely with agency polygons than FIRED. Averaged across all provinces, CFSDS^25^ has an index of 0.73 ± 0.31, while FIRED^16^ has an index of 0.47 ± 0.32 (Table 4, Fig. 4). This is likely due to the lower spatial resolution of FIRED^16^ and the temporal lag commonly associated with MODIS burned area products^12^.

**Table 4:** Provincial summary of Sørensen–Dice index of agency polygons compared against FIRED^16^ and CFSDS^25^ perimeters.

Province	FIRED vs Agency	CFSDS vs Agency	n
Alberta	0.46 ± 0.33	0.73 ± 0.32	98
British Columbia	0.49 ± 0.32	0.72 ± 0.32	442
Manitoba	0.42 ± 0.29	0.76 ± 0.26	73
Quebec	0.40 ± 0.33	0.77 ± 0.26	85
Saskatchewan	0.47 ± 0.35	0.72 ± 0.31	23
Average	0.47 ± 0.32	0.73 ± 0.31	721

Each sample represents a single day of fire spread for which an agency polygon is available. The reported average is weighted by growth day.

### Daily burned area correlation

**Fig. 5:**
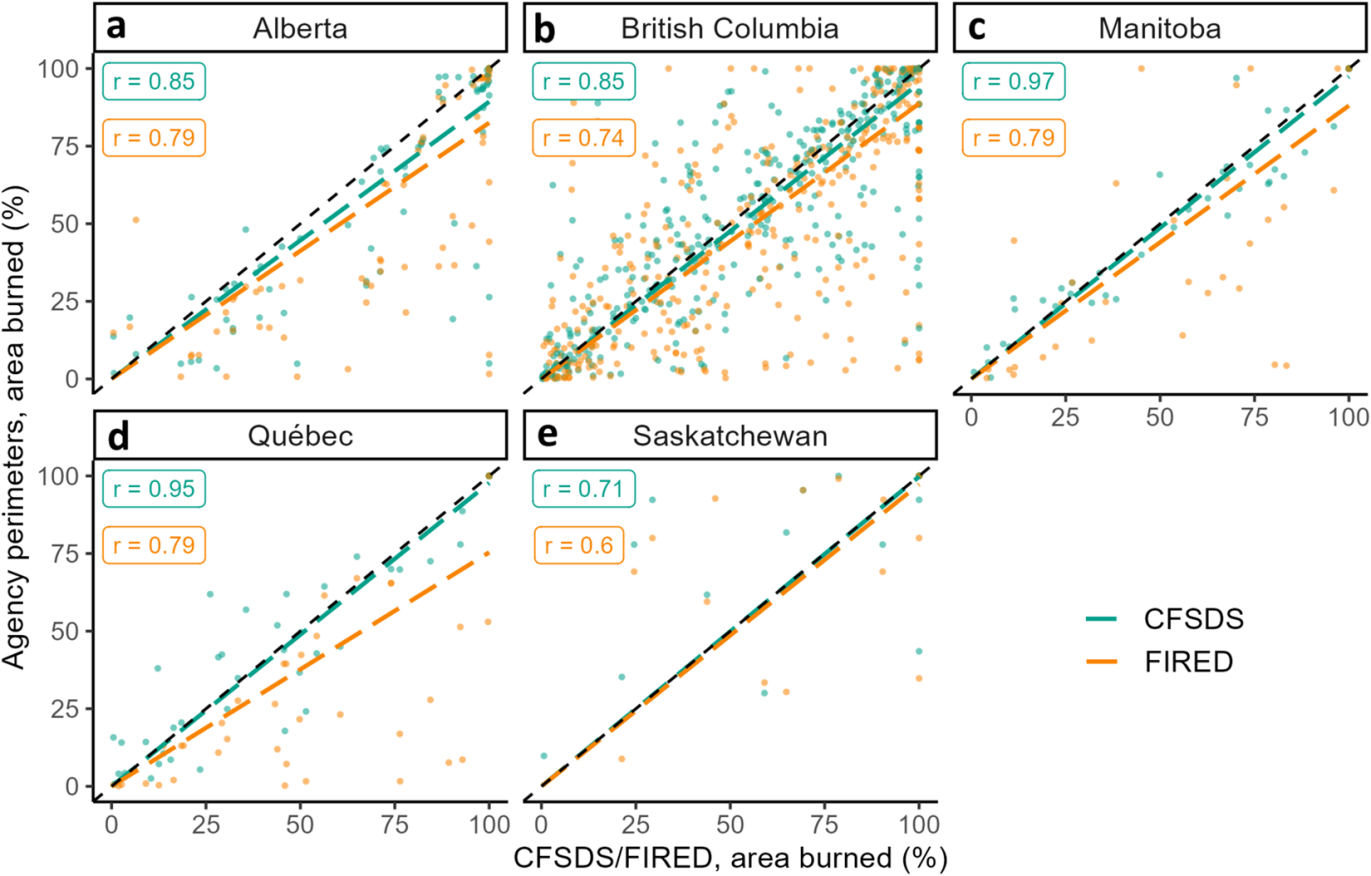
Daily area-burned validation by province. Agency polygons from the provincial wildfire agencies of Alberta (**a**), British Columbia (**b**), Manitoba (**c**), Quebec (**d**), and Saskatchewan (**e**) provide the validation dataset to compare against the CFSDS or FIRED. Points represent a single agency polygon burning day compared against FIRED or CFSDS using percentage burned of the final perimeter. Dashed black line represents 1:1 fit, or perfect agreement with agency polygons. Dashed, coloured lines represent a linear regression fit of all points. Spearman correlation coefficients are indicated with inset boxes.

Validation by comparing daily area burned is an established method for intercomparison of remotely sensed fire progressions against agency polygons^47^. We calculated the Spearman correlation between each dataset’s prediction of daily area burned for individual fires, showing that the CFSDS^25^ consistently outperforms FIRED (Fig. 5), with CFSDB having a Spearman’s ρ 0.06–0.28 higher than FIRED. On a daily basis, both datasets tended to underpredict total area burned when compared against the agency perimeters, although this could also be attributed to agency perimeters overpredicting daily area burned. While MODIS burned area products^12^, such as FIRED^16^, have been shown to be poorly suited for burned area assessments of smaller (<100 ha) fires^47^, our fire size threshold of 1,000 ha minimised this bias. It is notable that even with this advantage to FIRED^16^, CFSDS^25^ was the strongest performer.

## Usage Notes

Users interested in examining the spread of a specific fire or set of fires should identify the fire IDs of these fires using NBAC^26^, available at https://cwfis.cfs.nrcan.gc.ca/datamart/metadata/nbac. Individual fire events were assigned a unique ID by the concatenation of the ‘YEAR’ and ‘NFIREID’ fields from NBAC^26^. For example, a fire from 2007 with an NFIREID of 346 would have an ID of “2007_346” and a corresponding interpolation filename of “2007_346_krig.tif”.

CFSDS v1.0^25^ will be expanded annually with corresponding covariates as better data become available, and will be made available at the same data repository. (https://osf.io/f48ry/) (10.17605/OSF.IO/F48RY). This data descriptor was peer reviewed in 2024 on v1.0 of the dataset, and based on the data available at the time.

## Data Availability

R code used for producing CFSDS^25^ has been made available at the Centre for Open Science OSF data repository at https://osf.io/f48ry/ (10.17605/OSF.IO/F48RY). Four code sections are provided, grouped under one code file “CFSDS_example.R”. Code subsections: 1. ‘1.0 Processing’ - Preprocesses a single NBAC perimeter and FIRMS hotspots^9^. 2. ‘2.0 Interpolation’ - Interpolates fire arrival time for a single NBAC perimeter using kriging. 3. ‘3.0 Covariates - Uses the fire arrival time raster to extract environmental covariates associated with each burning day. 4. ‘4.0 Summarize’ - an example of how an end-user might summarize point-level spread data for use in fire modelling.
